# Prolonged survival after splenectomy in Wiskott-Aldrich syndrome: a case report

**DOI:** 10.1186/1824-7288-37-42

**Published:** 2011-09-10

**Authors:** Kostas N Syrigos, Nektaria Makrilia, Jeffrey Neidhart, Michael Moutsos, Sotirios Tsimpoukis, Maria Kiagia, Muhammad W Saif

**Affiliations:** 1 Oncology Unit, 3rd Department of Medicine, Sotiria General Hospital, Athens School of Medicine, Greece; 2 San Juan Oncology, New Mexico, USA; 3 Division of Hematology and Oncology, Department of Medicine, Columbia University, NY, USA

**Keywords:** Wiskott-Aldrich syndrome, thrombocytopenia, splenectomy, survival, treatment

## Abstract

Wiskott-Aldrich syndrome is a rare X-linked immunodeficiency disorder that is characterized by a variable clinical phenotype. Matched donor bone marrow transplantation is currently the only curative therapeutic option. We present the case of a 24-year-old male who was diagnosed at the age of seven with Wiskott-Aldrich syndrome. He did not respond to intravenous gammaglobulin and he experienced recurrent pulmonary infections despite prophylactic antibiotics. The patient had no matched donor. At the age of nine, he was submitted to splenectomy and his platelet count was normalized. Fifteen years later, the patient remains asymptomatic with a normal platelet count. He is still receiving prophylactic antibiotics and no bleeding episodes or septic complications have been reported. This case demonstrates that splenectomy can represent a safe therapeutic option in selected WAS patients, provided that there is a tight follow-up program, patient education and adherence to guidelines regarding post-splenectomy prophylaxis.

## Background

Wiskott-Aldrich syndrome (WAS) is a rare immunodeficiency disorder, which is characterized by recurrent infections, small-platelet thrombocytopenia and eczema. It has been associated with increased risk of autoimmune disorders and malignancy [1]. The syndrome was first described in 1937 by Wiskott in three brothers with congenital thrombocytopenia, eczema, bloody diarrhea and multiple ear infections [2], whereas, in 1954, Aldrich reported that this disorder is associated with X-linked inheritance [3]. WAS has a wide range of clinical phenotypes that correlate with the type of mutation in the *WASP *gene. The three main clinical phenotypes produced by *WASP *mutations are classic WAS, X-linked thrombocytopenia and X-linked neutropenia [4]. Clinical manifestations of WAS consist of petechiae, ecchymosis and bloody diarrhea [1]. Recurrent infections are a frequent problem and autoimmune diseases further complicate clinical presentation [4]. Treatment is mainly supportive and includes immunization, intravenous gammaglobulin, corticosteroids, transfusions, prophylactic antibiotics and splenectomy [5]. Matched bone marrow transplant seems to be the only curative therapeutic option [6], whereas gene therapy holds much promise for future use in clinical practice [7]. When a matched donor is not available, the number of treatment options available is small and overall prognosis is poor [6].

Splenectomy is used sparingly by WAS treatment centers, mainly because of the potential post-splenectomy complications [8]. We present the case of a patient with WAS with prolonged survival after splenectomy, who is on a tight follow-up program and tightly adheres to the prophylactic regimen. No sign of disease recurrence has been reported nor has any serious complication.

## Case presentation

### Case report

Our patient first presented at our department at the age of seven with facial eczema, recurrent pulmonary infections (lasting several weeks) and otitis media. Physical examination revealed a severe eczema and laboratory tests showed microcytic thrombocytopenia with a platelet count of 42000/mL at diagnosis. His white blood cell count and hemoglobulin were within normal range. Bone marrow biopsy ruled out aplastic anemia. He had a low IgG (495 mg/dl, normal levels > 560 mg/dl for age) and IgM (95 mg/dl, normal levels > 145 mg/dl for age) count and elevated IgA (270 mg/dl, normal levels < 250 mg/dl for age) and IgE levels (1680 IU/ml, normal levels < 420 IU/ml for age). Coagulation tests were normal as were the total count and morphology of megacaryocytes. He had no known family history of the disease but a maternal uncle was reported to have died in childhood after experiencing similar symptoms. The patient received multiple courses of intravenous gammaglobulin, steroids, transfusions and prophylactic antibiotics. His thrombocytopenia was refractory (platelet count ranging from 8000-78000/mL) and he experienced multiple bleeding episodes. At the age of eight, he also exhibited autoimmune Henoch-Schönlein-like purpura and lymphopenia of variable severity. The patient had neither a matched-donor sibling nor a matched unrelated donor. At the age of nine, the patient underwent splenectomy. His platelet count was normalized has been normal ever since with a mean value of 235000/mL. There have been no signs of disease recurrence in the last fifteen years or any autoimmune manifestations of WAS. Levels of immunoglobulins are within normal limits and the eczema has not reappeared. The patient was educated from a very young age about the importance of taking specific post-splenectomy preventive measures: Vaccination for pneumonococcus, haemophilus influenza B and meningococcal C pathogens is conducted according to guidelines and antibody levels are measured to make sure that response is adequate. The patient is currently on life-long phenoxymethylpenicillin 250 mg per os twice daily. All post-splenectomy febrile illnesses of the patient are carefully evaluated and complete blood work is performed to rule out sepsis. Antibiotic sensitivity testing on isolated pathogens is performed. A mutation analysis was conducted six years ago and a missence mutation in the exon 2 of the patient's WASP gene was detected. Furthermore, the patient lacked WASP protein expression. He has experienced no episodes of post-splenectomy bleeding or sepsis. His compliance with the recommended treatment has always been exceptional.

## Discussion

WAS is a multifaceted syndrome which is characterized by sinopulmonary infections, microthrombocytopenia and eczema [1]. The vast majority of patients have episodes of bleeding, which are life-threatening in a percentage reaching 30% [9]. According to the Zhu classification (1997) [10], our patient is classified as score 4 WAS, due to severe, treatment-resistant eczema, recurrent infections and presence of autoimmune disease (Henoch-Schönlein-like purpura). He also lacked WASP protein expression, which can be rarely observed in missence patients [11]. However, it should be noted that missence mutations to exons 1-3 have been associated with milder phenotypes [10] and positive WASP expression [11].

The mutations responsible occur in the WAS gene, that regulates actin polymerization in hematopoietic cells. Thrombocytopenia in this syndrome seems to be attributed to increased phagocytosis and spleen destruction as well as ineffective thrombocytopoiesis. Patients with WAS have combined immunodeficiency, as functions of T and B lymphocytes and NK cells are affected [4]. Main causes of death are severe bleeding episodes, infections, autoimmune diseases and hematological malignancies [1]. Currently, the only curative therapy for WAS is hematopoietic stem cell transplantation (HSCT) [6], whereas gene therapy is still under study [7]. Matched unrelated donors can also be used for HSCT in WAS patients. A 2006 Japanese study showed that the overall 5-year survival rate is 80% for WAS patients treated with HSCT from matched unrelated donors or unrelated cord blood [12], while an Italian single-center trial concluded that survival rates were even better (81.2%) in case of unrelated donors [13]. Transplant is, however, associated with significant risk of morbidity and mortality and this should be taken into account, especially in patients with some other phenotype variant and not classic WAS [6]. In patients with no available matched donor or ineligible because of age, treatment is mainly supportive with intravenous gammaglobulin, prophylactic antibiotics and immunization. Systemic steroids generally fail to improve thrombocytopenia and immunocompromise the patient even further. The outcome is usually poor in these patients with only few of them surviving into adulthood [8].

Splenectomy is one of the treatment options available in patients who are not submitted to transplantation but it is used sparingly by centers worldwide, since it carries significant risk of severe infections and sepsis [8]. Conley et al [8] in 2003 reported that less than 10% of treatment centers use splenectomy systematically as a treatment option whereas, 26.3% never select this modality. It has been shown to be an effective measure to sustain an increase in platelet counts [14]. The exact mechanism of action is not known, however, it is believed that platelet destruction is decreased and that blocking of this clearance mechanism assists in offsetting impaired platelet production [15]. The main concern regarding the wider use of splenectomy is the post-splenectomy bleeding and sepsis [14]. The Mullen et al study [14] showed that recurrence of bleeding was mainly reported in patients who developed idiopathic thrombopenic purpura (ITP) and that half of these patients had already been diagnosed with ITP before splenectomy. A recent multicenter study conducted in a large cohort of XLT patients showed that the overall cumulative incidence of serious bleeding events is not significantly reduced after splenectomy [5]. Regarding post-splenectomy sepsis, more than half of the patients were not receiving the recommended prophylactic antibiotics or had discontinued the regimen [14]. According to Albert et al [16], despite the higher risk for systemic infections after splenectomy, antibiotic prophylaxis and vaccination may render splenectomy a reasonable treatment of choice for some XLT patients, provided that the family situation and compliance issued are taken into careful account.

Our case confirms that prolonged survival can be achieved post-splenectomy in patients with WAS, given that there is a systematic approach in their post-splenectomy medical care. Contrary to historical experience, this patient experienced prolonged normalization of his platelet count after splenectomy. Increased survival after splenectomy has been previously reported but we propose a cautious and systematic post-operative surveillance which seems to improve the outcome. The patient had been very carefully educated regarding the importance of complying with the preventive regimen and of informing all treating physicians about his being asplenic before receiving any treatment. A careful and sensitive approach helped avoid compliance issues in adolescence as well, which has been recognized as a period of increased risk of treatment refusal [16]. The guidelines for prevention and treatment of infections in patients with absent or dysfunctional spleen were followed, as have been published and revised by the British Committee for Standards in Hematology [17]. Furthermore, the patient received the recommended immunization for splenectomized patients and the adequate response to the vaccination was monitored by regularly measuring antibody levels. It should be noted that the patient produces anti-polysaccharide antibodies at levels within normal range, even though a defective WAS gene has been detected. Improvement in cellular immunity has been described after splenectomy in WAS since 1981 [18] and this could be associated with the adequate response to vaccination observed in our case. Post-splenectomy, he did not experience increased infectious complication while on prophylactic antibiotics and routine vaccinations. Fifteen years after splenectomy, he continues to function as an energetic youth without complications. The appropriate timing of splenectomy and selection of candidates are matters for discussion. With the emergence of antibiotic resistant strains of pneumonococcus, it is possible that regular infusions of IVIG are also needed as prophylaxis in cases of patients who do not adequately respond to vaccination.

## Conclusions

In conclusion, prolonged survival with no thrombocytopenia recurrence can be achieved in WAS patients who undergo splenectomy, without any grave complications, with a focus on post-splenectomy patient care and compliance.

## Competing interests

The authors declare that they have no competing interests.

Sources of funding: None

Helsinki declaration: Conformed

Written informed consent was obtained from this patient for publication of this case report. A copy of the written consent is available for review by the Editor-in-Chief of this journal.

## Authors' contributions

KNS made a substantial contribution in conception and design and critically revised the manuscript. NM acquired the data and drafted the manuscript. JN analyzed and interpreted the data and co-drafted the manuscript. MM analyzed the data and critically revised the manuscript. ST interpreted the data and co-drafted the manuscript. MK analyzed the data and critically revised the manuscript. MWS made a substantial contribution in conception and design and critically revised the manuscript. All authors gave final approval of the version to be published.
